# Consultation

**Published:** 1876-06

**Authors:** 


*Consultations.*—We will here make a remark which we doubt not will be sanctioned by a majority of our professional readers. It asserts a fact which is highly discreditable to medical men. It is this, that in nine cases in ten consultations are not conducted in accordance with medical ethics, and result in disparaging the physician first in attendance; not unfrequently causing him to be supplanted by the party consulted, or otherwise injured in his professional reputation. So true is this, and so common, that few practitioners will call a consultation if it can possibly be avoided.

There are numerous ways in which confidence is destroyed toward the practitioner. Sometimes by suggesting a thorough and radical change of treatment, when such change may not actually be necessary. Sometimes the practitioner is highly complimented for his tenderness or for his nursing qualities, while his judgment is by indirection disparaged. If a young man, a patronizing air is often assumed toward him, and he is kindly admonished how to act, and that in the presence of the patients and friends. A knowing look, a slight shake of the head, or silence when inquiries are made in regard to the previous conduct of the case, may do the work. Sometimes the remark is dropped that it is *now* too late to do this or that. The writer once heard this remark made to the father of the patient, by the physician consulted: "If I could only have seen this case a little sooner."

Now, it is well known that consultations in critical cases are usually desired by the attending physician, not because a change of treatment is actually required, or because he is ignorant of the treatment proper to the case, but for the purpose of restoring the waning confidence of the friends, and in the event of death, to guard against any reflection by the friends upon the practitioner, or upon themselves, for neglect of the use of every available means, supposed or real, for the patient's recovery.

This being true, it is manifestly the duty, and should be the pleasure, of every medical man, when called in consultation, to guard with the greatest courtesy and caution the feelings and reputation of the brother with whom he is called to consult.

He should approve, if possible, what he has done, and by no means, directly or indirectly, do or say anything to lower him in the estimation of his patrons.

Brethren, the people care but little for us, except when they are sick. Let us not be untrue to ourselves. Our occupation is responsible, painful and trying at best. Let us not heighten our troubles by jealousies and unkindness toward each other. The golden rule is not less applicable to doctors than to other callings. "Do as you would be done by," should be our motto.

